# Autoimmune Responses to Soluble Aggregates of Amyloidogenic Proteins Involved in Neurodegenerative Diseases: Overlapping Aggregation Prone and Autoimmunogenic regions

**DOI:** 10.1038/srep22258

**Published:** 2016-02-29

**Authors:** Sandeep Kumar, A. Mary Thangakani, R. Nagarajan, Satish K. Singh, D. Velmurugan, M. Michael Gromiha

**Affiliations:** 1 Biotherapeutics Pharmaceutical Sciences, Pfizer Inc., 700 Chesterfield Parkway West, Chesterfield MO 63017, USA; 2 Center for Advanced Studies in Crystallography and Biophysics and Bioinformatics Infrastructure Facility, University of Madras, Chennai 600025, India; 3 Department of Biotechnology, Bhupat Jyoti Mehta School of Biosciences, Indian Institute of Technology Madras, Chennai 600036, India

## Abstract

Why do patients suffering from neurodegenerative diseases generate autoantibodies that selectively bind soluble aggregates of amyloidogenic proteins? Presently, molecular basis of interactions between the soluble aggregates and human immune system is unknown. By analyzing sequences of experimentally validated T-cell autoimmune epitopes, aggregating peptides, amyloidogenic proteins and randomly generated peptides, here we report overlapping regions that likely drive aggregation as well as generate autoantibodies against the aggregates. Sequence features, that make short peptides susceptible to aggregation, increase their incidence in human T-cell autoimmune epitopes by 4–6 times. Many epitopes are predicted to be significantly aggregation prone (aggregation propensities ≥10%) and the ones containing experimentally validated aggregating regions are enriched in hydrophobicity by 10–20%. Aggregate morphologies also influence Human Leukocyte Antigen (HLA) - types recognized by the aggregating regions containing epitopes. Most (88%) epitopes that contain amyloid fibril forming regions bind HLA-DR, while majority (63%) of those containing amorphous β-aggregating regions bind HLA-DQ. More than two-thirds (70%) of human amyloidogenic proteins contain overlapping regions that are simultaneously aggregation prone and auto-immunogenic. Such regions help clear soluble aggregates by generating selective autoantibodies against them. This can be harnessed for early diagnosis of proteinopathies and for drug/vaccine design against them.

Millions of people suffer from neurodegenerative diseases caused by deposition of amyloid plaques in the brain. During the early stages of these diseases, soluble aggregates of the amyloidogenic proteins often generate autoimmune responses among the patients^1^,^2^,^3^,^4^. Presently, it is not understood how and why the human immune system selectively engages with the soluble aggregates of amyloidogenic proteins. In order to investigate this question, we have analyzed available experimental data on molecular determinants of protein aggregation and autoimmunity.

Protein aggregation has been well studied in the literature^5^,^6^. It has been shown that typically 5–9 residues long sequence regions, called aggregation prone regions, are capable of forming cross-β motifs and nucleating aggregation^7^. Several computational methods are available to identify such regions in protein sequences^8^. Similarly, presentation of the Human Leukocyte Antigen (HLA) restricted peptides to the T-cell receptors is a crucial step in generation of T-cell mediated immune responses against an antigen^9^. These HLA restricted peptides are called T-cell immune epitopes and are often 9–16 residues long. Potential immune epitopes capable of binding to different HLA-loci and their subtypes can be also predicted computationally^10^,^11^,^12^. Along with the available experimental data, ability to predict both aggregation prone regions and T-cell immune epitopes in protein sequences can be used to understand how T-cell mediated autoimmune responses selectively target the soluble aggregates of human amyloidogenic proteins.

In this work, amino acid sequences of the experimentally validated human T-cell autoimmune epitopes, aggregating peptides and human amyloidogenic proteins were analyzed for overlap between aggregation prone regions and T-cell autoimmune epitopes. The experimental data, collected from the literature, was supplemented with randomly generated peptide sequences and computational predictions of aggregation prone regions as well as T-cell autoimmune epitopes. Our analyses reveal that overlapping aggregation prone and T-cell autoimmune epitope regions drive aggregation of the amyloidogenic proteins as well as generation of autoantibodies that selectively bind their aggregates. Generation of selective autoantibodies against the soluble aggregates sets them up for clearance from the body and helps maintain homeostasis.

## Results

### Description of Datasets

Detailed descriptions of database searches, collection and classification of data are available in Methods section and Table S1 in Supplementary Information summarizes all datasets analyzed in this work. Briefly, 3941 experimentally validated T-cell autoimmune epitopes that bind HLA-DP (155), HLA-DQ (543) and HLA-DR (3243) were collected by searching the Immune Epitope Database, IEDB (http://www.iedb.org/)^13^. A set of 430 peptides known to not bind HLAs^14^,^15^ (MHCIInonbind) was used as a control dataset. Experimentally validated datasets of 536 peptide sequences that form amyloid fibrils (Amyloid536) and 158 hexapeptides that form amorphous-β aggregates (Amor158)^16^ were used to scan the autoimmune epitopes for regions susceptible to aggregation. The Amyloid536 dataset includes a subset of 194 hexapeptides that form amyloid fibrils (AmyloidHexpep194), along with a set of 308 amyloid-fibril forming peptides that are longer than 6 residues and 34 experimentally studied peptides from amyloidogenic proteins. Three hundred and fifty nine hexapeptides that form β-stands (Globular359βstrands) in globular monomeric proteins were used as another control dataset. Further, we have also utilized forty four human amyloidogenic proteins and a dataset of 100,000 randomly generated 15-residues long peptide sequences for the analysis.

### Aggregating peptides in T-cell autoimmune epitopes

Sequence features that make short peptides prone to β-strand mediated aggregation also increase their incidence in the human T-cell autoimmune epitopes as shown in Table 1. Only 21 of the 359 (5.8%) β-strand forming hexapeptides from globular monomers (Globular359βstrands) are found in the autoimmune epitopes. In comparison, aggregating peptides from different datasets, Amor158, AmyloidHexpep194 and Amyloid536, are found in autoimmune epitopes with proportions of 23.7–39% (Table 1). These 4 to 6 fold increased incidences for the aggregating peptides are highly significant at 95% confidence level (p-value  < 0.05), as indicated by change of proportion test^17^. The incidences of amyloid-fibril forming peptides in the autoimmune epitopes are also significantly greater than those in MHCIInonbind, the dataset of peptides that do not bind HLAs (Table 1).

**Figure 1:**
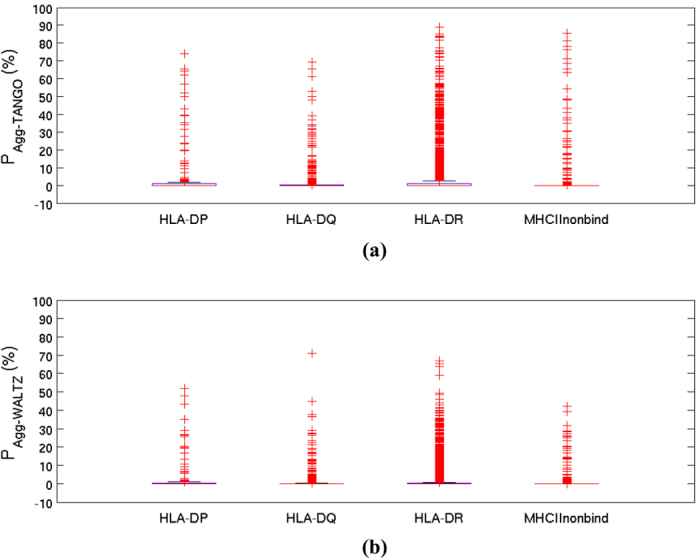
Box and Whisker plots showing average aggregation propensities of the peptide sequences in experimentally validated human T-cell autoimmune epitopes and MHCIInonbind datasets, predicted by (**a**) TANGO (P_Agg-TANGO_) and (**b**) WALTZ (P_Agg-WALTZ_). All epitope classes (HLA-DP, HLA-DQ and HLA-DR) show long tails suggesting that a number of epitopes in these datasets are aggregation prone.

Substantial proportions of the human T-cell epitopes are predicted to be aggregation prone. There is no experimental data available on aggregation behaviors of T-cell autoimmune epitopes in this study. Therefore, aggregation propensity, total aggregation score normalized by the number of residues in a sequence, of each T-cell autoimmune epitope was computed using TANGO^18^ and WALTZ^19^. The results are presented as Box and Whisker plots in Fig. 1 and average values are shown in Table S2. There is a wide scatter in the data and several epitopes, especially the HLA-DR binding ones, have aggregation propensities ≥10%. An aggregation propensity cut off value of 10% was arbitrarily chosen to identify significantly aggregation prone epitopes. In summary, regions that promote aggregation as well as HLA binding in peptide sequences appear to have common physicochemical properties.

### Aggregate morphology and autoimmune epitopes

Morphological features of aggregates, amorphous β-aggregates *versus* amyloid fibrils, are correlated with their relative incidences in the autoimmune epitopes (Table 1). Approximately 24% (46) of the hexapeptides that form amyloid fibrils occur in the autoimmune epitopes while only 3.6% (7) of such peptides are found in the control dataset, MHCIInonbind. This avoidance of MHCIInonbind by amyloid fibril forming hexapeptides is statistically significant at 95% confidence level (p-value < 0.05). When amyloid fibril forming peptides of all lengths (Amyloid536 dataset) are considered, 209 (39%) of such peptides are found in the autoimmune epitopes, while only 17 (3.2%) of them occur in MHCIInonbind. Of 341 amyloid fibril forming peptides that are longer than six residues, 163 (47.8%) are found in the autoimmune epitopes, while only 10 (2.9%) of them occur in MHCIInonbind (Table 1). In contrast to above findings, amorphous β-aggregating hexapeptides show no significant preferences to be found in either the autoimmune epitopes or MHCIInonbind. Forty nine (31%) of the 158 amorphous β-aggregating hexapeptides are found in the autoimmune epitopes and 39 (24.7%) of them are in MHCIInonbind. Only the exact matches were considered here because mutation of even a single residue can disrupt an aggregating sequence pattern^20^.

Aggregate morphology also influences HLA-loci bound by the aggregating peptide sequence region containing autoimmune epitopes. The HLA-DP binding autoimmune epitopes do not contain any experimentally validated aggregating sequence region (Table 1). Therefore, this analysis is limited to HLA-DQ and HLA-DR binding autoimmune epitopes. Figure S1 compares frequencies of HLA-DQ and HLA-DR binding autoimmune epitopes which contain amorphous β-aggregating or amyloid fibril forming sequence regions. HLA-DQ is preferentially recognized when the epitopes contain amorphous β-aggregating regions. Of 49 autoimmune epitopes that contain amorphous β-aggregating hexapeptides, 31 (63%) bind HLA-DQ and the remaining 18 bind HLA-DR (Table 1). In contrast, three-fourths (34, 74%) of the 46 epitopes that contain amyloid fibril forming hexapeptides regions bind HLA-DR and only 12 bind HLA-DQ. Most (184, 88%) of the 209 autoimmune epitopes, which contain amyloid fibril forming regions of all lengths, bind HLA-DR. Furthermore, of the 163 autoimmune epitopes that contain aggregating peptides longer than six residues, 150 (92%) bind HLA-DR (Table 1). The HLA subtypes within the same locus (HLA-DP, DQ or DR) show common sequence preferences for the repertoire of peptides they recognize^21^. Amyloid fibril forming and amorphous β-aggregating peptides also show analogous position dependent residue pair preferences among sequences of their own types^16^. Therefore, the above results suggest synergies may exist between sequence pattern requirements for different aggregate morphologies and HLA bindings.

### Aggregation prone T-cell autoimmune epitopes are enriched in hydrophobicity

Our previously reported simulations of peptide sequences showed that hydrophobicity may be a common factor between aggregation prone regions and HLA-DR binding T-cell immune epitopes^22^. In this work, Table 2 compares average and standard deviation values for intrinsic hydrophobicity of the sequences in different datasets of aggregating peptides and autoimmune epitopes, calculated using a scale developed by Hodges and coworkers^23^. The intrinsic hydrophobicity values have been normalized by peptide lengths to allow direct comparisons. The aggregating peptides, both amyloid fibril forming (38.0 ± 12.9 for 194 hexapeptides and 32.3 ± 11.2 for all 536 peptides) and amorphous β-aggregating (35.6 ± 13.2), have greater hydrophobicity than the autoimmune epitopes (28.4 ± 7.6 for all epitopes, 30.3 ± 8.9 for HLA-DP, 26.9 ± 7.3 for HLA-DQ and 28.5 ± 7.5 for HLA-DR binding epitopes). As a result, the autoimmune epitopes that contain aggregating peptide regions become more hydrophobic than those with no such regions. Table 2 shows that autoimmune epitopes which contain aggregating regions are enriched in hydrophobicity by 10–20%. For example, all epitopes that contain amyloid fibril forming hexapeptides have average hydrophobicity of 31.9 ± 4.0, an enrichment of 12%. Therefore, only small enrichments in intrinsic hydrophobicity are needed for T-cell autoimmune epitopes to accommodate aggregation prone regions. Although biologically meaningful, the enrichments in hydrophobicity of the autoimmune epitopes are not statistically significant. This is expected because the amino acid sequences of autoimmune epitopes, including those containing aggregation prone regions, are determined by binding site characteristics of their cognate HLAs^21^. This finding was confirmed by analyses of the randomly generated peptides (described later).

### Amyloidogenic proteins contain overlapping aggregation prone and T-cell autoimmune epitope regions

**Figure 2:**
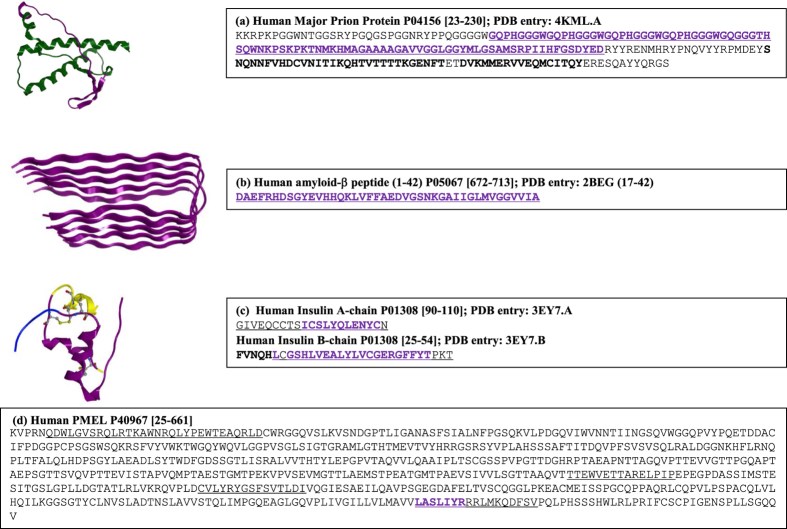
Overlap between aggregation prone and T-cell autoimmune epitope regions in human amyloidogenic proteins. Human amyloidogenic proteins whose amino acid sequences contain experimentally validated aggregating peptides (bold font) as well as experimentally validated T-cell autoimmune epitope peptides (underlined) are shown. All available experimental data on these proteins is consolidated into the regions highlighted in the sequences. The PDB^26^ was queried for available structural information on these proteins and information obtained was used to map overlapping aggregation prone and autoimmune epitope regions in the respective protein structures. These regions are shown in magenta. The magenta regions in structural images correspond to the sequence regions that are simultaneously shown in bold magenta font and underlined. (**a**) Human Major Prion Protein. Structural information is from the PDB entry 4 KML chain A. The N-terminal residues 1–116 are not present in the structure. (**b**) Human Amyloid-β peptide 1–42. Structural information is from the PDB entry 2BEG, which shows this peptide in fibrillary form. N-terminal residues 1–16 are not present in the structure. (**c**) Human Insulin. Structural information was taken from the PDB entry 3EY7. The chains A and B are shown in yellow and blue ribbons. The disulfide bonds are also shown. (**d**) Human Pmel. No structural information is available for this protein.

The experimentally validated T-cell autoimmune epitopes studied in this report belong to several different types of proteins. However, almost all of the epitopes that contain experimentally validated aggregating peptide sequences are from the amyloidogenic proteins. These observations suggest that human amyloidogenic proteins may contain overlapping aggregation prone and T-cell autoimmune epitope regions in their amino acid sequences. To further explore this possibility, peptide sequences in the datasets of experimentally validated autoimmune epitopes and aggregating peptides were scanned for incidence among 44 human amyloidogenic proteins. This analysis yielded exact matches in 206, 57, 26 and 5 instances with the sequences of amyloid-fibril forming peptides, amorphous β-aggregating hexapeptides, HLA-DR and HLA-DQ binding T-cell autoimmune epitopes, respectively. Again, only the identical matches were used here. Like the aggregation prone regions, single point mutations can also disrupt sequence patterns required for binding HLAs and/or antigen processing^24^. The 26 HLA-DR binding autoimmune epitopes belong to Major Prion Protein, Amyloid β-peptide, Insulin, Pmel and Fibrinogen-α, and all the 5 HLA-DQ binding autoimmune epitopes belong to Insulin. The 26 HLA-DR epitopes overlap with 96 amyloid-fibril forming peptides and 15 amorphous β-aggregating peptides. Similarly, the 5 HLA-DQ binding autoimmune epitopes detected in human Insulin overlap with 15 amyloid fibril forming peptides and 17 amorphous β-aggregating peptides. All the experimentally validated overlapping aggregation prone and T-cell autoimmune epitope regions belong to major prion protein, Amyloid β peptide, Insulin and Pmel. Figure 2 highlights these regions in their sequences and structures. In case of Amyloid β peptide and Insulin, such overlapping regions span almost entire sequences. Insulin hormone has been also developed into several therapeutic products and aggregates present in these products have been linked to undesirable immune responses, such as injection site reactions, among some patients^25^. A crystal structure of HLA-DQ: Insulin B-chain (11-LVEALYLVCGERGG-24) complex is available in the Protein Data Bank (PDB)^26^ entry 1JK8. The peptide in this complex contains an amyloid-fibril forming portion 11-LVEALYL-17 whose crystal structures in fibrillary forms are also present in the PDB entries 3HYD and 3OMQ. In case of human major prion protein, the overlapping aggregation prone and autoimmune epitope regions traverse a large portion of disordered sequence at the N-terminus, while portions of the C-terminal region are aggregation prone but are not part of T-cell autoimmune epitopes. Interestingly, Pmel which forms functional amyloids^19^ also shows an overlap between an experimentally validated T-cell autoimmune epitope and an aggregating peptide (Fig. 2). This observation is consistent with ability of human cells to tolerate protein aggregates for functional benefit^27^.

To supplement the experimental evidence described above, predictions for T-cell autoimmune epitopes were carried out on amino acid sequences of the amyloidogenic proteins using a reference set of HLA alleles for maximum population coverage^28^ available in the IEDB^13^. Only the very strongly predicted T-cell autoimmune epitopes (percentile cut off ≤1) were selected for further analyses to assure high confidence that most of the predicted T-cell autoimmune epitopes would indeed bind HLAs, when tested experimentally. There are 48 HLA-DP, 68 HLA-DQ and 169 HLA-DR binding potential T-cell autoimmune epitopes (285 in total) in the amyloidogenic proteins, with several of them overlapping with one another. A majority (155 out of 285, 54.4%) of the predicted epitopes are promiscuous and can bind more than one HLA-loci (see Table S3). In particular, most of the HLA-DP and HLA-DQ binding epitopes are promiscuous. For example, 28 of the 48 (58.3%) predicted HLA-DP binding epitopes can also bind to HLA-DR and 13 (27.1%) of them can bind all HLA-types. In contrast to HLA-DP and HLA-DQ binding epitopes, most of the 169 predicted HLA-DR binding epitopes are fidel and 102 of them (60%) bind only to HLA-DR. Here, the concept of fidelity is applied at the level of major HLA loci, DP, DQ or DR. However, each HLA locus is degenerate due to genetic polymorphism and a large number of HLA subtypes exist in human populations^21^.

**Figure 3:**
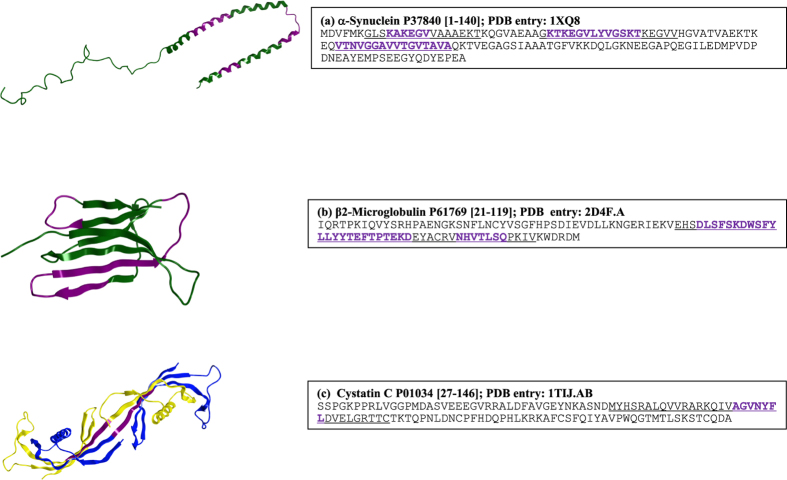
Additional examples of overlap between aggregation prone and T-cell autoimmune epitope regions in human amyloidogenic proteins are presented. This figure is prepared in the same way as the Fig. 2, except that the T-cell autoimmune epitope shown here are the strongly predicted ones. (**a**) Human α-Synuclein. Structural information is from the PDB entry 1XQ8. This structure is for the micelle bound form of α-Synuclein. In the unbound form, it is an intrinsically disordered protein. (**b**) Human β2-microglobulin. Structural information is from the PDB entry 2D4F. (**c**) Human Cystatin C. The structural information is from the PDB entry 1TIJ chains A and B. This PDB entry contains domain swapped form of Cystatin C dimer. Both the chains are shown here in yellow and blue ribbons.

In the next step, the 285 strongly predicted T-cell autoimmune epitopes were clustered. The 155 promiscuous T-cell autoimmune epitopes overlapped with one another and were combined into 64 regions. None of the remaining 130 fidel epitopes overlapped with one another. This yielded a total of 194 autoimmune epitope regions in amyloidogenic protein sequences. These regions were scanned with experimentally validated aggregating peptides. Thirty three (11.6%) of them were found to contain at least one aggregating peptide sequence region. Nineteen of these 33 epitope regions are fidel and the remaining 14 are promiscuous. Table 3 presents all the 33 regions along with the names of source proteins and associated disease information, obtained primarily from UniProt^29^. Figure 3 shows the overlaps between predicted T-cell Immune epitopes (all HLA-loci combined) with experimentally validated aggregating peptides in amino acid sequences and three dimensional structures of α-synuclein (micelle bound form), α2-microglobulin and Cystatin C, respectively. The overlapping aggregation prone and T-cell autoimmune epitope regions span different secondary structure types; α-helices in micelle bound α-synuclein, α-strands and coil regions in α2-microglobulin and α-strands in Cystatin C. This observation can be rationalized by noting that the locations as well as conformations of these regions will most likely change in the aggregation competent states of these proteins. In particular, α-synuclein is an intrinsically disordered protein whose extreme conformational flexibility has earned it the name ‘protein-chameleon’^30^. The amino acid sequence of α-synuclein contains three strongly predicted T-cell autoimmune epitopes and all of them contain experimentally validated aggregating peptides. This observation is consistent with the presence of multiple autoantibodies against soluble aggregates of α-synuclein in blood sera of patients suffering from Parkinson’s disease^1^,^3^.

To further probe the overlap between T-cell autoimmune epitopes and aggregation prone regions in the amyloidogenic proteins, potential aggregation prone regions were also predicted in the amyloidogenic protein sequences using TANGO^18^ and WALTZ^19^. Figure S2 in Supplementary Information shows all predicted aggregation prone regions and their overlap with the strongly predicted fidel and promiscuous T-cell autoimmune epitope regions in the amyloidogenic protein sequences. More than two-thirds (31 out of 44, 70.5%) of the amyloidogenic proteins in this study are predicted to contain at least one overlapping aggregation prone and autoimmune epitope region. Aggregation propensities of the 194 strongly predicted T-cell autoimmune epitope regions in amyloidogenic proteins were also computed, and their box and whisker plots are shown in Figure S3. Consistent with the findings on the experimentally validated T-cell autoimmune epitopes, several strongly predicted epitopes also show significant aggregation propensities.

### Overlap between aggregation prone regions and T-cell autoimmune epitopes in 100,000 randomly generated peptide sequences

One hundred thousand 15-residues long peptides of non-identical sequences were randomly generated to study the overlap between T-cell autoimmune epitopes and aggregation prone regions *via* mathematical simulations. These peptides have same amino acid composition as the dataset of 3,243 HLA-DR binding T-cell autoimmune epitopes. HLA-DR binding T-cell autoimmune epitopes are the largest set of experimentally validated autoimmune epitopes in this study and these epitopes contain aggregating peptides with the greatest frequencies (Table 1). Length of each randomly generated peptide was chosen to be 15 residues because this is the most frequent peptide length for the HLA-DR binding epitopes. All the 100,000 peptides were subjected to predictions for T-cell autoimmune epitopes and aggregation prone regions.

Sequence patterning is an important determinant for presence of T-cell autoimmune epitopes and aggregation prone regions in the randomly generated peptide sequences. Of the 100,000 randomly generated peptides, only 16,384 (16.4%) are predicted to be T-cell autoimmune epitopes. Similarly, 12,179 (12.2%) peptides are predicted to contain at least one TANGO predicted aggregation prone region and 9,582 (9.6%) peptides are predicted to contain one or more WALTZ predicted aggregation prone region (Table 4). Among the 16,384 T-cell autoimmune epitopes, 3,620 (22.1%) also contain TANGO predicted aggregation prone regions. If the sequence patterning requirements for aggregation prone regions and T-cell autoimmune epitopes were unrelated, then only 1,995 (16,384 × (12,179/100,000) = 1995.41) epitopes are expected to contain such regions. This leads to a preference value of 1.8 (3,620/1,995) for the T-cell autoimmune epitopes to contain TANGO predicted aggregation prone regions. Similarly, 2,321 of the 16,384 T-cell autoimmune epitopes (14.2%) contain WALTZ predicted aggregation prone regions as compared to the expected 1,570, yielding a preference value of 1.5 (Table 4). Both of these statistical preferences are highly significant at the 95% level of confidence (p-value <0.05), as shown by change in proportion tests.

The availability of random peptides with same amino acid composition as the HLA-DR binding T-cell autoimmune epitopes allows for further probe into the enrichment of intrinsic hydrophobicity for the autoimmune epitopes that contain aggregation prone regions. Table S4 compares average intrinsic hydrophobicity values for the random peptides in different subsets. The average intrinsic hydrophobicity for all random peptides is 28.4 ± 7.5. This is similar to the average intrinsic hydrophobicity (28.5 ± 7.5) for 3,243 experimentally validated HLA-DR binding autoimmune epitopes (Table 2). The average intrinsic hydrophobicity for the 16,384 predicted T-cell autoimmune epitopes is 32.7 ± 7.0 (median = 32.5). This increase of approximately 15% is statistically insignificant, since it falls within one standard deviation of the average intrinsic hydrophobicity for all random peptides. The average intrinsic hydrophobicity for the 3,620 T-cell autoimmune epitopes whose sequences contain TANGO predicted aggregation prone regions is 38.0 ± 6.4. This is an additional enrichment of 16% over the average intrinsic hydrophobicity for all T-cell autoimmune epitopes. Similarly, the 2,321 autoimmune epitopes which contain WALTZ predicted aggregation prone regions are further enriched by approximately 8%, with their average intrinsic hydrophobicity being 35.1 ± 6.3. Like the experimentally validated T-cell autoimmune epitopes (Table 2), the increases in average values of intrinsic hydrophobicity for the randomly generated T-cell autoimmune epitopes that contain predicted aggregation prone regions are statistically insignificant. These observations suggest that incidence of aggregation prone regions within the T-cell autoimmune epitopes does not require any significant sequence composition adjustments.

The aggregation propensities for the randomly generated peptides were also evaluated and the results, shown in Figure S4 and Table S4, are similar to those obtained from analyses of the experimental data (Fig. 1). The average aggregation propensity computed by TANGO for all randomly generated peptides is 4.8 ± 12.2 (median = 0). The large standard deviation value over the mean is consistent with large scatter in the data. The T-cell autoimmune epitopes have average TANGO aggregation propensity value of 9.3 ± 16.8 (median = 0.2) and the average TANGO aggregation propensity for the 3,620 autoimmune epitopes that contain TANGO predicted aggregation prone regions is 34.8 ± 18.0 (median = 34.7). The trend in the aggregation predictions performed using WALTZ is similar to that described above.

**Figure 4:**
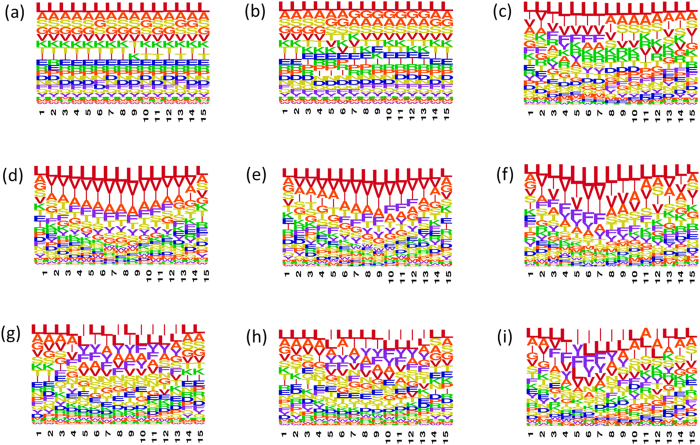
Sequence patterning synergies between the T-cell autoimmune epitopes and aggregation prone regions. Sequence logos are shown for (**a**) 100,000 randomly generated 15-residues long peptides, (**b**) 83,616 random peptides that were not predicted to be T-cell autoimmune epitopes (non-epitopes), (**c**) 16,384 strongly predicted T-cell autoimmune epitopes, (**d**) 12,179 random peptides that contain TANGO predicted APRs, (**e**) 8,559 non-epitopes that contain TANGO APRs, (**f**) 3,620 T-cell autoimmune epitopes that contain TANGO APRs, (**g**) 9,582 random peptides that contain WALTZ predicted APRs, (**h**) 7,261 non-epitopes that contain WALTZ APRs, and (**i**) 2,331 T-cell autoimmune epitopes that contain WALTZ APRs. Note the enrichment of hydrophobic β-branched and aromatic residues, particularly Val, Ile, and Phe, at initial positions of T-cell autoimmune epitope peptides (see panels **c,f,i**).

Finally, uniform peptide length facilitated construction of sequence logos for the randomly generated peptides and their various subsets (Fig. 4). It is well known that aggregating regions in the amyloidogenic proteins are enriched in hydrophobic β-branched and aromatic residues. Consistently, the random and non-epitope peptides (83,616 peptides that are not predicted to be T-cell autoimmune epitopes) with TANGO and WALTZ predicted aggregation prone regions are rich in such residues at most positions (Fig. 4(d,e,g,h)). Figure 4(c) shows that the initial positions, 1–7, in the 16,384 strongly predicted T-cell autoimmune epitopes are also rich in such residues, particularly, in Val, Ile and Phe. These sequence patterning synergies at the initial positions are further enhanced for T-cell autoimmune epitopes that contain TANGO and WALTZ predicted aggregation prone regions (see Fig. 4(f,i)).

## Discussion

Maintenance of homeostasis is a complex task that involves multiple cellular pathways^31^,^32^,^33^,^34^. Aging diminishes ability of the cells to maintain proteostasis and neurons are particularly vulnerable to diseases caused by protein aggregation^31^. Formation of protein aggregates is a common feature among neurodegenerative diseases^32^,^33^ and autoantibodies reactive to the soluble aggregates of several amyloidogenic proteins have been reported in the literature^1^,^2^,^3^,^35^. Detection of autoantibodies against soluble aggregates of α-synuclein in blood sera of the patients with Parkinson’s disease has been proposed as a diagnostic test^1^. A therapeutic antibody aducanumab, which targets small aggregates of amyloid-β at early stages of Alzheimer’s disease, is in clinical trials^36^. Despite these advances, molecular basis of selective autoimmune responses against the soluble aggregates of amyloidogenic proteins has remained unexplored. Several datasets of aggregating peptides, T-cell autoimmune epitopes, amyloidogenic proteins and randomly generated peptides were analyzed here to explore potential molecular origins of this phenomenon. The results consistently suggest that aggregation prone regions can overlap with T-cell autoimmune epitopes. The evidence in favor of the overlap is available at two levels. First, sequences of experimentally validated aggregating peptides prefer to occur within those of experimentally validated human T-cell autoimmune epitopes. Second, full length sequences of several amyloidogenic proteins contain overlapping aggregation prone and autoimmune epitope regions. Furthermore, analyses of the 100,000 randomly generated 15-residues long peptide sequences also show highly significant preferences for T-cell autoimmune epitopes to contain aggregation prone regions. The overlapping aggregation prone and auto-immunogenic regions can potentially drive aggregation as well as generation of selective autoantibodies against the aggregates. It should be clarified here that not all aggregation prone regions fall within T-cell autoimmune epitopes and majority but not all of the amyloidogenic proteins contain above described overlapping regions. This is expected because both autoimmunity and aggregation play many different physiological roles. The randomly generated peptide sequences further validate this point (Table 4).

We hypothesize that the overlap between aggregation prone and T-cell autoimmune epitope regions allows human immune system to selectively engage with the protein aggregates and set them up for clearance. Clearance of aggregates contributes towards maintenance of homeostasis which is an important physiological requirement, especially in the context of aging. Putatively, this is a protective feature of humoral immunity, in addition but unrelated to natural tolerance towards self-antigens. Most aggregation prone regions are solvent inaccessible in the native state of a protein, being buried in structural scaffoldings, and are therefore inactive^37^,^38^. However in misfolded aggregation competent conformations, some of these regions may become solvent exposed and actively promote aggregation. The aggregates need to be soluble in humoral fluids and small enough for uptake by the antigen presenting cells as well as for renal clearance. In agreement with this argument, Ghiso *et al*.^39^ have reported presence of soluble amyloid β aggregates as normal component of urine and Takata *et al*.^40^ have proposed detection of amyloid β in the urine samples of patients suffering with Alzheimer’s disease and mild cognitive impairment as a non-invasive biomarker for diagnostic and monitoring purposes. Overall, this hypothesis provides a potential explanation for generation of autoimmune responses among patients at the early stages of neurodegeneration. Promiscuous binding of aggregation prone T-cell autoimmune epitopes by multiple HLAs can ensure that autoantibodies against such aggregates are generated in broad populations. In agreement with this hypothesis, current literature contains several experimental reports on individual monoclonal antibodies shown to bind oligomers of amyloidogenic proteins and interfere with their aggregation process. Notably, certain anti-α-synuclein antibodies can inhibit aggregation of α-synuclein even when antibody to protein/aggregate stoichiometric ratio is very low^41^. Furthermore, antibody mediated clearance of α-synuclein aggregates from extracellular regions has been shown to prevent inter-cellular transmission of these aggregates^42^. In another example consistent with the above hypothesis, Asante and coworkers^43^ have recently reported that a naturally occurring variant of major prion protein (G127 V) provides protection from prion diseases in humans. This mutation falls within the N-terminal overlapping aggregation prone and T-cell autoimmune epitope region of the major human prion protein (see Fig. 2).

The mechanism that underpins selective binding of the soluble protein aggregates by autoantibodies needs to be experimentally studied in molecular details. The overall scheme may be analogous to the one that has been proposed to explain immunogenicity arising from aggregates present in the biologic drug products^44^,^45^. Briefly, the T-cell dependent immune response requires uptake of protein aggregates by antigen presenting cells for digestion into peptides which bind HLAs and are presented on the surfaces of these cells (see Fig. 3 in Kumar *et al*.^45^). Recognition of HLA: peptide complex by T-cell receptors is essential for activation of T-cells, along with the presence of appropriate cytokine signals. This series of steps ultimately results in production of IgG autoantibodies, capable of selectively binding aggregates, by the plasma cells. The amyloidogenic proteins studied here contain multiple overlapping T-cell autoimmune epitopes and aggregation prone regions. Soluble aggregates of an amyloidogenic protein can be thought of as an ensemble of different oligomeric species presenting different autoimmune epitopes. In agreement with these, multiple autoantibodies are generated against the soluble aggregates of α-synuclein by patients suffering from Parkinson’s disease^1^,^3^.

The overall incidence of experimentally validated aggregating peptides in a given class of human T-cell autoimmune epitopes is low, as expected (Tables 1 and 4). In spite of the intrinsic hydrophobicity and sequence patterning synergies between aggregation prone regions and T-cell autoimmune epitopes, protein aggregation is only one of the several factors that can lead to autoimmune responses. Moreover, several aggregating peptides in Amyloid536 dataset are longer than the T-cell epitopes. The analyses performed here were also supplemented by the predictions of aggregation prone regions and MHCII T-cell immune epitopes. Performances of all prediction algorithms need to be continuously validated and improved to assure correct predictions. To maximize the likelihood that the predictions are true, the T-cell autoimmune epitopes in amyloidogenic proteins were identified by using IEDB recommended method^46^ and a very strong cut off percentile value (percentile ≤1) was used. The aggregation prone region prediction programs, TANGO and WALTZ, have been also extensively experimentally validated by their developers^18^,^19^,^47^. In spite of these efforts, it is conceivable that a few of the overlaps between T-cell autoimmune epitopes and aggregation prone regions in the amyloidogenic protein sequences (Figure S2) may be artefacts of the predictions.

An overlap between aggregation prone and T-cell autoimmune epitope regions may be necessary but not sufficient for production of IgG autoantibodies against the aggregates. Some T-cell autoimmune epitopes may be the regulatory ones (Tregitopes) and suppress rather than enhance autoimmune responses against the aggregates^48^. The ability of aggregation prone regions to actively promote aggregation is also modulated by sequence and structural contexts they are located in^38^,^49^. Analogous overlaps among three dimensional aggregation prone motifs and B-cell epitopes which may facilitate T-cell independent adaptive immune responses were not studied here. Potential interactions of protein aggregates with receptors that may lead to activation of innate immune response^50^ were also not studied in this work.

In spite of the above limitations, this analysis is a first step towards understanding molecular origins and purpose of the selective autoimmune responses against soluble aggregates of the amyloidogenic proteins. As more experimental data becomes available in the literature and our abilities to identify aggregation prone regions and immune epitopes *in silico* improve, additional examples corroborating a role for protein aggregation in autoimmune diseases will emerge. It is anticipated that additional research will build on this foundation and facilitate early diagnosis of protein aggregation diseases. It will also contribute towards novel drug and T-cell epitope driven vaccine design^51^ opportunities for treatment of proteinopathies as well as for the early diagnosis of the neurodegenerative diseases.

## Methods

### Datasets used in this study

Table S1 summarizes general characteristics of all the datasets used in this study.

#### Human T-cell Auto Immune Epitopes

Experimentally validated sets of T-cell autoimmune epitopes that bind HLA-DP, HLA-DQ and HLA-DR were collected by searching the Immune Epitope Database, IEDB^13^ (http://www.iedb.org/), for all *Homo sapiens* MHC II binding T-cell immune epitopes in June 2014. The search results were clustered at 100% sequence identity and overlapping epitopes were combined into one. Only the human autoimmune epitopes with non-identical sequences were taken. This yielded 155 HLA-DP, 543 HLA-DQ and 3243 HLA-DR binding T-cell autoimmune epitope sequences. A set of peptides known to not bind human HLA^14^,^15^ were also used (MHCIInonbind) as a control dataset.

#### Aggregating Peptides

Experimentally validated datasets of 536 peptide sequences of all lengths that have been shown to form amyloid fibrils (Amyloid536) and 158 hexapeptides that form amorphous β aggregates (Amor158)^16^ were used to scan the human T-cell autoimmune epitopes for regions susceptible to aggregation. The Amyloid536 peptides include a subset of 194 hexapeptides experimentally shown to form amyloid fibrils (AmyloidHexpep194), 308 peptides that are 7–72 residues long and have been experimentally shown to form amyloid fibrils, and 34 experimentally studied peptides from amyloidogenic proteins^16^. 33 of these 34 peptides are also more than 6 residues long (7–141 residues). Therefore, the total number of amyloid fibril forming peptides longer than 6 residues in Amyloid536 dataset is 341. 359 hexapeptide sequences that form β-stands in globular monomeric proteins were used as a control dataset (globular359βstrands). All the globular monomeric proteins are non-amyloidogenic and most (68%) of the 359 hexapeptides are solvent inaccessible in the context of their parent protein structures. Overall, the aggregating peptides in different datasets and the control β-strand peptides can be found in 195 unique proteins.

#### Human Amyloidogenic Proteins

All human amyloidogenic proteins including those that form the functional amyloids were included in this study. The sequences for a majority of these proteins were taken from Tsolis *et al*.^52^. These were supplemented with human protein sequences in UniProt (www.uniprot.org) annotated as amyloidogenic. Again only the proteins with non-identical sequences were retained. This dataset contains 44 human proteins including 11 amyloidogenic light chains. The amino acid sequences of some amyloidogenic light chains are highly homologous. All the homologues were retained because even a single residue mutation can potentially disrupt an aggregation prone region or a T-cell autoimmune epitope or both.

#### One hundred thousand 15-Residues Long Peptide sequences

One hundred thousand (100,000) peptide sequences of length 15 residues were randomly generated such that they have the same amino acid composition as the 3,243 HLA-DR binding T-cell autoimmune epitopes. These peptide sequences were subjected for predictions for T-cell Immune epitopes and aggregation prone regions, as described below. This dataset supplements the datasets of experimentally validated T-cell autoimmune epitopes and aggregating peptides, and allows for further evaluation of overlap between aggregation prone regions and T-cell autoimmune epitopes, using larger numbers of sequences. Two of the 100,000 randomly generated peptide sequences contain short peptides from the experimentally validated HLA-DR binding T-cell autoimmune epitopes. None but two of the 16,384 predicted T-cell autoimmune epitopes among these randomly generated peptides overlap with experimentally validated aggregating peptide sequences.

### Overlapping aggregating peptides and T-cell autoimmune epitopes

Amino acid sequences of amyloidogenic proteins and experimentally validated human T-cell autoimmune epitopes were scanned for incidence of experimentally validated aggregating peptides. The amyloidogenic protein sequences were also scanned for incidence of experimentally validated T-cell autoimmune epitopes. In all cases, only the identical matches were recorded. All the matches were consolidated into aggregating and autoimmune epitope regions and the overlaps were noted by examining individual sequences. Note that an autoimmune epitope or an amyloidogenic protein can contain several aggregating peptides. Similarly, an aggregating peptide can occur in more than one autoimmune epitope. Two or more aggregating peptides may also overlap.

### Prediction of aggregation prone regions and T-cell autoimmune epitopes

To supplement the experimental observations, computer algorithms capable of identifying potential aggregation prone regions and MHC II binding T-cell immune epitopes were used. Total aggregation scores for each sequence belonging to experimentally validated human T-cell autoimmune epitope and amyloidogenic protein datasets were calculated using TANGO (http://tango.crg.es/)^18^ and WALTZ (http://waltz.switchlab.org/)^19^. All the calculations were performed at temperature 298 K, pH 7.4, ionic strength 0.150 molar and concentration of 1 millimolar. Potential aggregation prone regions, of lengths ≥6 residues and aggregation score ≥10%, were also identified in these sequences. A minimum length of six residues was chosen because hexapeptides and longer peptides have been often studied for aggregation in experiments. TANGO uses statistical mechanics to identify general β-strand mediated aggregation prone regions in protein and peptide sequences. The TANGO predicted aggregation prone regions may produce aggregates with different morphologies, ranging from amorphous aggregates to amyloid fibrils. WALTZ, on the other hand, uses position specific matrices to identify amyloid fibril forming APRs. Therefore, we are able to predict both amyloid-fibril forming and amorphous-β aggregating aggregation prone regions by combining these programs. Moreover, these programs have been and are being continuously validated by the authors *via* experiments^18^,^19^,^47^ and are being incorporated into protein solubility improvement protocols^53^. Aggregation propensity of a sequence was calculated by normalizing total aggregation score for the sequence by number of residues in the sequence^54^.

The T-cell autoimmune epitope predictions were carried out using IEDB recommended method (http://tools.iedb.org/mhcii/)^46^ for all HLA alleles in the reference set for maximum population coverage^28^. As described by Wang *et al*.^46^, IEDB recommended method is a consensus approach that combines predictions from at least three of the four leading T-cell autoimmune epitope prediction programs (also see help pages on IEDB website for further details). Only the strong binders, those with ≤1 percentile score, were selected. The selected autoimmune epitopes were clustered and mapped onto the amyloidogenic protein sequences.

### Average intrinsic hydrophobicity

All sequences in the datasets containing experimentally validated aggregating peptides, human T-cell autoimmune epitopes and randomly generated peptides were used for calculation of intrinsic hydrophobicity using a scale described by Hodges and coworkers^23^. Intrinsic hydrophobicity (H) of a given peptide sequence was computed using the following equation:


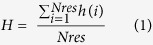


where *h(i)* is the hydrophobicity coefficient of *i*^*th*^ residue and *Nres* is the number of residues in the peptide sequence.

### Change in Proportion test

Change in proportion tests were performed as described earlier^17^,^55^. This test was used to determine statistical significance (at 95% level of confidence, p-value <0.05) of the changes in incidences of the peptides from Globular359βstrands, Amor158, AmyloidHexpep194 and Amyloid536 datasets within the peptides from MHCIInonbind and human T-cell autoimmune epitopes. This test was also used to test significance of overlaps between potential aggregation prone regions and T-cell autoimmune epitopes in 100,000 randomly generated peptides.

### Generation of Sequence Logos

Sequence logos were generated for 100,000 randomly generated peptides and their various subsets using Seqlogo function in MATLAB and enoLOGOS web server^59^.

## Additional Information

**How to cite this article**: Kumar, S. *et al*. Autoimmune Responses to Soluble Aggregates of Amyloidogenic Proteins Involved in Neurodegenerative Diseases: Overlapping Aggregation Prone and Autoimmunogenic regions. *Sci. Rep.* **6**, 22258; doi: 10.1038/srep22258 (2016).

## Supplementary Material

Supplementary Information

## Figures and Tables

**Table 1:** Incidence of experimentally validated aggregating peptides within the experimentally validated human T-cell autoimmune epitopes.

	359 β-strandforming hexapeptides from globular proteins	158 amorphous β-aggregatesforming hexapeptides	194 amyloid fibrilforming hexapeptides	341 amyloid fibrilforming peptides of length 7–141	536 amyloid fibrilforming peptides of all lengths
155 HLA-DP bindingAutoimmune epitopes	0	0	0	0	0
543 HLA-DQ bindingAutoimmune epitopes	0	31 (19.6%)	12 (6.2%)	13 (3.8%)	25 (4.7%)
3243 HLA-DR bindingAutoimmune epitopes	21 (5.8%)	18 (11.4%)	34 (17.5%)	150 (44%)	184 (34.3%)
All 3941 T-cellautoimmune epitopes	21 (5.8%)	49 (31%)	46 (23.7%)	163 (47.8%)	209 (39%)
430 MHCIInonbindpeptides	0	39 (24.7%)	7 (3.6%)	10 (2.9%)	17 (3.2%)

Percentages were calculated with respect to the number of aggregating peptides in each column.

**Table 2:** Enrichment in hydrophobicity for human T-cell autoimmune epitopes that contain aggregating peptides.

Dataset	Average Hydrophobicity
All amorphous β-aggregates forming hexapeptides	35.6 ± 13.2
All amyloid fibril forming hexapeptides	38.0 ± 12.9
All amyloid fibril forming peptides of all lengths	32.3 ± 11.2
All human T-cell autoimmune epitopes	28.4 ± 7.6
All human T-cell autoimmune epitopes that contain amorphous β-aggregating hexapeptides	31.3 ± 4.3
All human T-cell autoimmune epitopes that contain amyloid fibril forming hexapeptides	31.9 ± 4.0
All human T-cell autoimmune epitopes that contain amyloid fibril forming peptides of all lengths	30.5 ± 4.4
All HLA-DP binding epitopes	30.3 ± 8.9
All HLA-DQ binding epitopes	26.9 ± 7.3
HLA-DQ binding epitopes that contain amorphous β-aggregating hexapeptides	31.3 ± 3.8
HLA-DQ binding epitopes that contain amyloid fibril forming hexapeptides	32.4 ± 3.3
HLA-DQ binding epitopes that contain amyloid fibril forming peptides of all lengths	31.8 ± 3.1
All HLA-DR binding epitopes	28.5 ± 7.5
HLA-DR binding epitopes that contain amorphous β-aggregating hexapeptides	31.3 ± 5.3
HLA-DR binding epitopes that contain amyloid fibril forming hexapeptides	31.6 ± 4.5
HLA-DR binding epitopes that contain amyloid fibril forming peptides of all lengths	30.1 ± 4.7

Average and standard deviation values of intrinsic hydrophobicity of peptide sequences in a given dataset of aggregating peptides and epitopes were computed as described in Methods.

**Table 3:** Strongly predicted T-cell autoimmune epitope regions in human amyloidogenic proteins that contain experimentally validated aggregating peptides.

Protein name, UniProt ID and Disease	T-cell autoimmune epitope region(s)
Apolipoprotein A-I,P02647 [25–267], Amyloidosis 8	219-LPVLESFKVSFLS**ALEEYT**KK-239
Apolipoprotein C-II,P02655 [23–101],Hyperlipoproteinemia 1B	48-KLRDLYSK**STAAMSTYTGIFTDQVLSVLK**GEE-79
α-Synuclein P37840[1–140] Parkinsondisease 1	7-GLS**KAKEGV**VAAAEKT-2231-G**KTKEGVLYVGSKT**KEGVV-4963-**VTNVGGAVVTGVTAVA**-78
β2-Microglobulin,P61769 [21–119],Hypercatabolic hypoproteinemia	55-EHS**DLSFSKDWSFYLLYYTEFTPTEKDE**Y-78
β2-Microglobulin,P61769 [21–119],Hypercatabolic hypoproteinemia	79-ACRV**NHVTLSQ**PKIV-93
Calcitonin, P01258 [85–116],Medullary carcinomaof thyroid^56^	13-TQ**DFNKFH**TFPQTAIGVGA-31
Cystatin-C,P01034 [27–146],Amyloidosis 6	41-MYHSRALQVVRARKQIV**AGVNYFL**DVELGRTTC-73
Gelsolin P06396 [28–782],Amyloidosis 5	183-**FNNGDCFILDLGNNI**HQWCGSNSNRYERLKATQVSKGIRD-222
IAPP (Amylin) P10997 [34–70],Type 2 diabetes	17-V**HSSNNFGAILSSTNVGSNTY**-37
Insulin B-chain P01308 [25–54],Diabetes mellitus,Injection localized amyloidosis^56^	5-HLCGSH**LVEALYLVC**GER-22
Kerato-epithelin,Q15582 [24–683],Corneal dystrophy	462-RGRYGTLFTMDRVLTPPMGTVMDVLKGDNR**FSMLVAAIQSA**GLTET-507
Lactoferrin, P02788 [20–710],Familial subepithelial cornealamyloidosis^57^	538-**NAGDVAFV**KDVTVLQNTDGNN-558
Lung Surfactant Protein C,P11686 [24–58],Pulmonary surfactant metabolism dysfunction 2	6-CPV**HLKRLLIVVVVVVLIVVVIVGALLMG**L-35
Lysozyme C, P61626 [19–148],Amyloidosis 8	4-E**RCELARTLKR**LGMDGYRGIS**LANWMCLAKW**ESGYNT-4051-STDYG**IFQINS**RYWCNDGK-69
Major Prion Protein,P04156 [23–230],Creutzfeldt-Jakob disease (CJD)	81-SKPKTN**MKHMAGAAAAGAVV**GGLG**GYMLGS**AMSRPII-117147-Y**SNQNNFVHDCVNITIK**QHTV-167
Pmel, P40967 [25–661]	74-PD**GQVIWVN**NTIINGSQV-91378-P**AEVSIVV**LSGTTAAQVTTTEWVETTARELPIP-410570-GQVPLIVGILLVLMAVV**LASLIYR**RRLMKQDF-601
Natriuretic peptides B,P16860 [27–134],Isolated Atrial Amyloidosis^58^	55-EVATEGIRGHR**KMVLYTL**RAPRSPKMV-81
Transthyretin, P02766 [21–147], Hyperthyroxinemia, dystransthyretinemic, Carpal tunnel syndrome 1	9-K**CPLMVKVLDAV**RGSP-24101-GPRR**YTIAALLSPYS**YSTTAVVTNPK-126

Strongly predicted (percentile ≤1.0) T-cell autoimmune epitopes in each amyloidogenic protein are overlapping and were therefore clubbed into T-cell autoimmune epitope regions. These regions were then searched for the incidence of experimentally validated aggregating peptides and are shown in bold fonts. The protein sequence codes and associated disease names were primarily taken from UniProt^29^. In the cases where this information is not available in UniProt, the disease names were taken from literature searches and citations are provided in this table.

**Table 4:** Predicted T-cell autoimmune epitopes and aggregation prone regions in 100,000 randomly generated 15-residues long peptides.

Sequences	Actual number andfrequency of TANGOpredicted APRs	Expected number andfrequency of TANGOpredicted APRs	Preference	Actual number andfrequency of WALTZpredicted APRs	Expected number andfrequency of WALTZpredicted APRs	Preference
100,000 Randomly generated Peptides	12,179 (12.2%)	NA	NA	9,582 (9.6%)	NA	NA
16,384 peptides predicted T-cell autoimmune epitopes	3,620 (22.1%)	1,995 (12.2%)	1.8	2,321 (14.2%)	1,570 (9.6%)	1.5
Remaining 83,616 peptides	8,559 (10.2%)	10,184 (12.2%)	0.8	7,261 (8.7%)	8,012 (9.6%)	0.9

The 100,000 randomly generated peptides have the same amino acid composition as 3,243 experimentally validated HLA-DR binding T-cell autoimmune epitopes. The incidences of TANGO and WALTZ predicted aggregation prone regions in all 100,000 randomly generated peptides were used to compute the expected incidences of TANGO and WALTZ predicted aggregation prone regions within 16,384 predicted T-cell autoimmune epitopes. In each case, the expected number of aggregation prone regions was rounded off to its closest integer value. Preference was computed by dividing the actual numbers of TANGO or WALTZ predicted aggregation prone regions in the 16,384 autoimmune epitopes by their respective expected numbers. Similar calculations were also performed for the remaining 83,616 peptides that do not meet the percentile cut off used in this study (see methods for more details). NA stands for not applicable.
