# Supplementary material for: Autoimmune Responses to Soluble Aggregates of Amyloidogenic Proteins Involved in Neurodegenerative Diseases: Overlapping Aggregation Prone and Autoimmunogenic regions
Source: Sci Rep. 2016 Feb 29;6:22258. doi: 10.1038/srep22258 (PMC4770294; doi:10.1038/srep22258)
Supplement: Supplementary Information [file srep22258-s1.pdf]

Autoimmune Responses to Soluble Aggregates of Amyloidogenic Proteins Involved in Neurodegenerative Diseases: Overlapping Aggregation Prone and Autoimmunogenic regions

Sandeep Kumar[1,*], A. Mary Thangakani[2], R. Nagarajan[3], Satish K. Singh[1], D. Velmurugan[2] and M. Michael Gromiha[3,*]

[1]Biotherapeutics Pharmaceutical Sciences, Pfizer Inc., 700 Chesterfield Parkway West, Chesterfield, MO 63017, USA

[2]Center for Advanced Studies in Crystallography and Biophysics and Bioinformatics Infrastructure Facility, University of Madras, Chennai 600025, India

[3]Department of Biotechnology, Bhupat Jyoti Mehta School of Biosciences, Indian Institute of Technology Madras, Chennai 600036, India

*Corresponding authors: Sandeep Kumar, AA411C, 700 West Chesterfield Parkway, Chesterfield 63017, MO, USA. Phone: +1-636-329-2362. Email: Sandeep.Kumar@pfizer.com and M. Michael Gromiha, Department of Biotechnology, Bhupat Jyoti Mehta School of Biosciences, Indian Institute of Technology Madras, Chennai 600036, India. Phone: +91-44-2257-4138. Email: gromiha@iitm.ac.in

Supplementary Information

Table S1. Average sequence lengths for peptides contained in experimentally validated human T-cell autoimmune epitopes, aggregating peptides and control datasets*

Dataset	Number	Average length	Median length	Range
HLA-DP	155	16.24±5.12	15	8-43
HLA-DQ	543	16.51±3.98	16	8-33
HLA-DR	3243	16.69±4.29	16	5-58
MHCIInonbind	430	15.74±3.17	15	6-25
Amyloid536	536	15.27±13.99	10	6-141
AmyloidHexpep194	194	6±0	6	6-6
Amor158	158	6±0	6	6-6
Globular359βstrands	359	6±0	6	6-6
100,000 randomly generated peptide sequences	100,000	15	15	15

*Data on human T-cell autoimmune epitopes were obtained from IEDB. The epitopes were divided into classes according to HLA locus bound, namely, HLA-DP, HLA-DQ and HLA-DR. MHCIInonbind stands for 430 peptides that do not bind human MHCII. Among the aggregating peptides, Amor158 stands for 158 hexapeptides that form amorphous β-aggregates, Amyloid536 stands for 536 peptides of different lengths that form amyloid fibrils, AmyloidHexpep194 is a subset of Amyloid536 and stands for 194 hexapeptides that form amyloid fibrils, and Globular359βstrands stands for 359 hexapeptides that form β-strands in monomeric globular proteins. MHCIInonbind and Globular359βstrands were used as control datasets in this study. See methods for details.

Table S2. Average aggregation propensities of human T-cell autoimmune epitopes*

Property	HLA-DP	HLA-DQ	HLA-DR	MHCIInonbind
Number of Epitopes	155	543	3243	430
Mean of Aggregation propensities calculated using TANGO	5.62±14.66	2.85±9.06	4.31±11.64	3.33±12.21
Epitopes with TANGO Aggregation Propensity ≥ 10%	22 (14.2%)	46 (8.5%)	394 (12.2%)	33 (7.7%)
Mean of Aggregation propensities calculated using WALTZ	3.16±8.85	1.52±5.88	2.06±6.51	1.51±5.76
Epitopes with WALTZ Aggregation Propensity ≥ 10%	16 (10.3%)	28 (5.2%)	227 (7.0%)	24 (5.6%)

*Aggregation propensity of a human T-cell autoimmune epitope was computed as sum of TANGO (or WALTZ) aggregation scores for all residues in the epitope divided by number of residues in the epitope. These aggregation propensities were then averaged for all epitopes in a class.

Table S3. Fidelity *versus* promiscuity in strongly predicted T-cell autoimmune epitopes in human amyloidogenic proteins*

HLA type	Number (%)
HLA-DP (Total)	48 (100)
HLA-DP (Fidel)	4 (8.3)
HLA-DP and HLA-DQ (Promiscuous)	3 (6.25)
HLA-DP and HLA-DR (Promiscuous)	28 (58.3)
HLA-DP, HLA-DQ and HLA-DR (Promiscuous)	13 (27.1)
HLA-DQ (Total)	68 (100)
HLA-DQ (Fidel)	24 (35.3)
HLA-DQ and HLA-DP (Promiscuous)	3 (4.4)
HLA-DQ and HLA-DR (Promiscuous)	28 (41.2)
HLA-DQ, HLA-DP and HLA-DR (Promiscuous)	13 (19.1)
HLA-DR (Total)	169 (100)
HLA-DR (Fidel)	102 (60)
HLA-DR and HLA-DP (Promiscuous)	23 (13.6)
HLA-DR and HLA-DQ (Promiscuous)	28 (16.6)
HLA-DR, HLA-DP and HLA-DQ (Promiscuous)	16 (9.5)

*Potential T-cell autoimmune epitopes binding to each HLA-locus were predicted for 44 human amyloidogenic protein sequences using the tools available at IEDB. Only very strongly predicted epitopes (percentile score ≤ 1.0) were included in this analysis. The word 'Total' in parentheses next to a given HLA-locus (DP, DQ or DR) indicates the total number of T-cell auto immune epitopes that are predicted to bind that HLA type. The word 'Fidel' in parenthesis next to a given HLA-locus (DP, DQ or DR) indicates the number of T-cell auto immune epitopes predicted to bind *only* that HLA-locus. By corollary, 'Promiscuous' indicates the number of T-cell autoimmune epitopes that are predicted to bind more than one HLA-locus. Evidence for promiscuity was gathered by scanning the peptides predicted to bind one HLA-type on to another HLA-locus. For example, HLA-DP and HLA-DR, indicates scanning of HLA-DP peptides on HLA-DR.

Table S4. Average aggregation propensity and intrinsic hydrophobicity for 100,000 randomly generated 15-residues long peptide sequences*

Random peptide sequence dataset	Average $P_{Agg\text{-}TANGO}$ (Median value)	Average $P_{Agg\text{-}WALTZ}$ (Median value)	Average H (Median value)
All 100,000 random peptides	4.78±12.21 (0)	2.25±6.95 (0)	28.37±7.52 (28.10)
16,384 peptides predicted to be T-cell autoimmune epitopes	9.35±16.83 (0.2)	3.21±8.15 (0.03)	32.67±7.04 (32.46)
Remaining 83,616 random peptides that are not predicted to be T-cell autoimmune epitopes (non-epitopes)	3.88±10.86 (0)	2.06±6.68 (0)	27.53±7.32 (27.21)
12,179 random peptides that contain TANGO predicted APRs	31.58±17.04 (31.45)		36.28±6.43 (36.02)
3,620 T-cell autoimmune epitopes that contain TANGO predicted APRs	34.85±18.04 (34.71)		38.03±6.41 (37.81)
8,559 non-epitopes that contain TANGO predicted APRs	30.19±16.40 (30.00)		35.54±6.30 (35.23)
9,582 random peptides that contain WALTZ predicted APRs		18.87±11.51 (16.20)	32.89±6.72 (32.69)
2,321 T-cell autoimmune epitopes that contain WALTZ predicted APRs		18.89±11.68 (16.26)	35.15±6.35 (34.76)
7,261 non-epitopes that contain WALTZ predicted APRs		18.87±11.46 (16.17)	32.17±6.68 (31.97)

*Average $P_{Agg\text{-}TANGO}$ stands for average aggregation propensity predicted by TANGO. Average $P_{Agg\text{-}WALTZ}$ stands for average aggregation propensity predicted by WALTZ. The aggregation propensity of a sequence was calculated by normalizing total aggregation score for the sequence by number of residues in the sequence. Average H stands for average intrinsic hydrophobicity. APR stands for Aggregation Prone Region. The median values are given in parentheses.

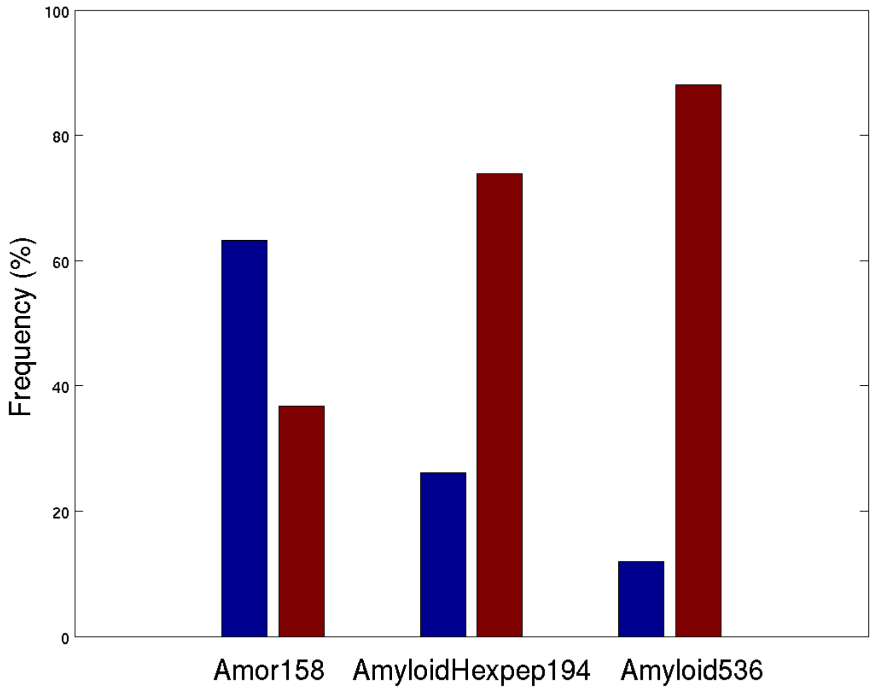

Figure S1: Aggregate morphologies influence incidence of aggregating peptides in experimentally validated T-cell autoimmune epitopes. Hexapeptides that form amorphous β aggregates (Amor158) occur with greater frequency in HLA-DQ binding T-cell autoimmune epitopes (blue Bars). In contrast, Amyloid-fibril forming peptides (AmyloidHexpep194 and Amyloid536) occur with greater frequencies in HLA-DR binding T-cell autoimmune epitopes (dark red bars).

Amyloidogenic protein sequences with predicted aggregation prone and T-cell autoimmune epitope regions

>Human Prolactin P01236 [29-227]
LPICPGGAARCQVTLRDLFDRAVV**LSHYIHNLSSEMFSEFDKRY**THGRGFITKAINSCHTSSLATPEDKEQAQ**QMNQKDFLSLIVS**
ILRSWNEPLYHLVTEVRGMQEAPEAILSKAVEIEEQTKRLLEGMELIVSQVHPETKENEIYPVWSGLPSLQMADEE**SRLSAYYNLL**
HCLRRDSHKIDNYLKLLKCRIIHNNNC

>Human Calcitonin P01258 [85-116]
CGNLSTCMLGTYTQDFNKFHTFPQTAIGVGAP

>Human Apolipoprotein A-I P02647 [25-267]
DEPPQS**PWDRVKDLATVYVDVLKDSGRDYVS**QFEGSALGKQLNLKLLDNWDSVTSTFSKLREQLGPVTQEFWDNLEKETEGLRQEM
SKDLEEVKAKVQPYLDDFQKKWQEEMELYRQKVEPLRAELQEGARQKLHELQEKLSPLGEEMRDRARAHVDALRTHLAPYSDELRQ
RLAARLEALKENGGARLAEYHAKATEHLSTLSEKAKPALEDLRQGL**LPVLESFKVSFLSALEEYTKK**LNTQ

>Human Serum Amyloid A P02735 [19-122]
RSFFSFLGEAFDGARDMWRAYSDMREANYIGSDKYFHARGNYDAAKRGPGGVWAAEAISDARENIQRFFGHGAEDSLADQAANEWG
RSGKDPNHFRPAGLPEKY

>Human Transthyretin P02766 [21-147]
GPTGTGESKCPLMVKVLDAVRGSPAINVAVHVFRKAADDTWEPFASGKTSESGELHGLTTEEEFVEGIYKVEIDTKSYWKALGISP
FHEHAEVVFTANDSGPRRYTIAALLSPYSYSTTAVVTNPKE

>Human Lactoferrin P02788 [20-710]
GRRRSVQWCAVSQPEATKCFQWQRNMRKVRGPPVSCIKRDSPIQCIQAIAENRADAVTLDGGFIYEAGLAPYKLRPVAAEVYGTER
QPRTHYYAVAVVKKGGSFQLNELQGLKSCHTGLRRTAGWNVPIGTLRPFLNWTGPPEPIEAAVARFFSASCVPGADKGQFPNLCRL
CAGTGENKCAFSSQEPYFSYSGAFKCLRDGAGDVAF**IRESTVFEDLSDEAERDE**YELLCPDNTRKPVDKFKDCHLARVPSHAVVAR
SVNGKEDAIWNLLRQAQEKFGKDKSPKFQLFGSPSGQKDLLFKDSAIGFSRVPPRIDSGLYLGSGYFTAIQNLRKSEEEVAARRAR
VVWCAVGEQELRKCNQWSGLSEGSVTCSSASTTEDCIALVLKGEADAMSLDGGYVYTAGKCGLVPVLAENYKSQQSSDPDPNCVDR
PVEGYLAVAVVRRSDTSLTWNSVKGKKSCHTAVDRTAGWNIPMGLLFNQTGSCKFDEYFSQSCAPGSDPRSNLCALCIGDEQGENK
CVPNSNERYYGYTGAFRCLAENAGDVAFVKDVTVLQNTDGNNNEAWAKDLKLADFALLCLDGKRKPVTEARSCHLAMAPNHAVVSR
MDKVERLKQVLLHQQAKFGRNGSDCPDKFCLFQSETKNLLFNDNTECLARLHGKTTYEKYLGPQYVAGITNLKKCSTSPLLEACEF
LRK

>Human Major Prion Protein P04156 [23-230]
KKRPKPGGWNTGGSRYPGQGSPGGNRYPPQGGGGWGQPHGGGWGQPHGGGWGQPHGGGWGQPHGGGWGQGGGTHSQWNKP**SKPKTN**
MKHMAGAAAAGAVVGGLGGYMLGSAMSRPIIHFGSDYEDRYYRENMHRYPNQVYYRPMDEYSNQNNFVHDCVNITIKQHTVTTTTK
GENFTETDVKMMERVVEQMCITQYERESQAYYQRGS

>Human Semenogelin I P04279 [24-462]
QKGGSKGRLPSEFSQFPHGQKGQHYSGQKGKQQTESKGSFSIQYTYHVDANDHDQSRKSQQYDLNALHKTTKSQRHLGGSQQLLHN
KQEGRDHDKSKGHFHRVVIHHKGGKAHRGTQNPSQDQGNSPSGKGISSQYSNTEERLWVHGLSKEQTSVSGAQKGRKQGGSQSSYV
LQTEELVANKQQRETKNSHQNKGHYQNVVEVREEHSSKVQTSLCPAHQDKL**QHGSKDIFSTQDELLVYNKNQ**HQTKNLNQDQQHGR
KANKISYQSSSTEERRLHYGENGVQKDVSQSSIYSQTEEKAQGKSQKQITIPSQEQEHSQKANKISYQSSSTEERRLHYGENGVQK
DVSQRSIYSQTEKLVAGKSQIQAPNPKQEPWHGENAKGESGQSTNREQDLLSHEQKGRHQ**HGSHGGLDIVIIEQEDDSDRHLAQHL**
NNDRNPLFT

>Human Beta-amyloid protein 42 P05067 [672-713]
DAEFRHDSGYEVHHQKLVFFAEDVGSNKGAIIGLMVGGVVIA

>Human Gelsolin P06396 [28-782]
ATASRGASQAGAPQGRVPEARPNSMVVEHPEFLKAGKEPGLQIWRVEKFDLVPVPT**NLYGDFFTGDAYVILKTVQLRNGNLQYDLH**
YWLGNECSQDESGAAAIFTVQLDDYLNGRAVQ**HREVQGFESATFLGYFKSGLKYKKGGVASGFKHVVPNEVVVQRLFQVKGRRVVR**
ATEVPVSWESFNNGDCFILDLGNNIHQWCGSNSNRYERLKATQVSKGIRDNERSGRARVHVSEEGTEPEAMLQVLGPKPALPAGTE
DTAKEDAAN**RKLAKLYKVSNGAGTMSVSLVADENPFAQGALKSEDCFILDHGKDGKIFVW**KGKQANTEERKAALKTASDFITKMDY
PKQTQVSVLPEGGETPLFKQFFKNWRDPDQTDGLGLSYLSSHIANVERV**PFDAATLHTSTAMAAQHGMD**DDGTGQKQIWRIEGSNK
VPVDPATYGQFYGGDSYIILYNYRHGGRQGQIIYNWQGAQSTQDEVAASAILTAQLDEELGGTPVQSRVVQGKEPAHLMSLFGGKP
MIIYKGGTSREGGQTAPASTRLFQVRANSAGATRAVEVLPKAGALNSNDAFVLKTPSAAYLWVGTGASEAEKTGAQELLRVLRAQP
VQVAEGSEPDGFWEALGGKAAYRTSPRLKDKKMDAHPPRLFACS**NKIGRFVIEEVPGELMQEDLATDDVMLLDTWDQV**FVWVGKDS
QEEEKTEALTSAKRYIETDPANRDRRTPITVVKQGF**EPPSFVGWFLGWDDDYWSVDPLDRAMAE**LAA

>Human Tau P10636 [2-758]
AEPRQEFEVMEDHAGTYGLGDRKDQGGYTMHQDQEGDTDAGLKESPLQTPTEDGSEEPGSETSDAKSTPTAEDVTAPLVDEGAPGK
QAAAQPHTEIPEGTTAEEAGIGDTPSLEDEAAGHVTQEPESGKVVQEGFLREPGPPGLSHQLMSGMPGAPLLPEGPREATRQPSGT
GPEDTEGGRHAPELLKHQLLGDLHQEGPPLKGAGGKERPGSKEEVDEDRDVDESSPQDSPPSKASPAQDGRPPQTAAREATSIPGF
PAEGAIPLPVDFLSKVSTEIPASEPDGPSVGRAKGQDAPLEFTFHVEITPNVQKEQAHSEEHLGRAAFPGAPGEGPEARGPSLGED
TKEADLPEPSEKQPAAAPRGKPVSRVPQLKARMVSKSKDGTGSDDKKAKTSTRSSAKTLKNRPCLSPKHPTPGSSDPLIQPSSPAV
CPEPPSSPKYVSSVTSRTGSSGAKEMKLKGADGKTKIATPRGAAPPGQKGQANATRIPAKTPPAPKTPPSSGEPPKSGDRSGYSSP
GSPGTPGSRSRTPSLPTPPTREPKKVAVVRTPPKSPSSAKSRLQTAPVPMPDLKNVKSKIGSTENLKHQPGGGKVQIINKKLDLSN
VQSKCGSKDNIKHVPGGGSVQIVYKPVDLSKVTSKCGSLGNIHHKPGGGQVEVKSEKLDFKDRVQSKIGSLDNITHVPGGGNKKIE
THKLTFRENAKAKTDHGAEIVYKSPVVSGDTSPRHLSNVSSTGSIDMVDSPQLATLADEVSASLAKQGL

>Human IAPP (Amylin) P10997 [34-70]
KCNTATCATQRLANFLVHSSNNFGAILSSTNVGSNTY

>Human Lung Surfactant Protein C P11686 [24-58]
FGIPC**CPVHLKRLLIVVVVVVLIVVVIVGALLMGL**

>Human alpha-Synuclein P37840 [1-140]
MDVFMKGLSKAKEGVVAAAEKTKQGVAEAAGKTKEGVLYVGSKTKEGVVHGVATVAEKTKEQVTNVGGAVVTGVTAVAQKTVEGAG
SIAAATGFVKKDQLGKNEEGAPQEGILEDMPVDPDNEAYEMPSEEGYQDYEPEA

>Human Lysozyme C P61626 [19-148]
KVFERCELARTLKRLGMDGYRGISLANWMCLAKWESGYNTRATNYNAGDRSTDYGIFQINSRYWCNDGKTPGAVNACHLSCSALLQ
DNIADAVACAKRVVRDPQGIRAWVAWRNRCQNRDVRQYVQGCGV

>Human beta2-Microglobulin P61769 [21-119]
IQRTPKIQVYSRHPAENGKSNFLNCYVSGFHPSDIEVDLLKNGERIEKV**EHSDLSFSKDWSFYLLYYTEFTPTEKDEY**ACRVNHVT
LSQPKIVKWDRDM

>Human Medin Q08431 [268-317]
RLDKQGNFNAWVAGSYGNDQWLQVDLGSSKEVTGIITQGARNFGSVQFVA

>Human Natriuretic peptides B P16860 [27-134]
HPLGSPGSASDLETSGLQEQRNHLQGKLSELQVEQTSLEPLQESPRPTGVWKSREVATEGIRGHRKMVLYTLRAPRSPKMVQGSGC
FGRKMDRISSSSGLGCKVLRRH

>Human Apolipoprotein C-II P02655 [23-101]
TQQPQQDEMPSPTFLTQVKESLSSYWESAKTAAQNLYEKTYLPAVDEKLRDLYSKSTAAMSTYTGIFTDQVLSVLKGEE

>Human ODAM A1E959 [16-279]
APLIPQRLMSASNSNELLLNLNNGQLLPLQLQGPLNSWIPPFSGILQQQQQAQIPGLSQFSLSALDQFAGLLPNQIPLTGEASFAQ
GAQAGQVDPLQLQTPPQTQPG**PSHVMPYVFSFKMPQEQGQMFQYYPVYMVLPWE**QPQQTVPRSPQQTRQQQYEEQIPFYAQFGYIP
QLAEPAISGGQQQLAFDPQLGTAPEIAVMSTGEEIPYLQKEAINFRHDSAGVFMPSTSPKPSTTNVFTSAVDQTITPELPEEKDKT
DSLREP

>Human Cystatin C P01034 [27-146]
SSPGKPPRLVGGPMDASVEEEGVRRALDFAVGEYNKASND**MYHSRALQVVRARKQIVAGVNYFLDVELGRTTC**TKTQPNLDNCPFH
DQPHLKRKAFCSFQIYAVPWQGTMTLSKSTCQDA

>Human Insulin B-chain P01308 [25-54]
FVNQHLCGSHLVEALYLVCGERGFFYTPKT

>Human Insulin A-chain P01308 [90-110]
GIVEQCCTSICSLYQLENYCN

>Human Acylphosphatase-2 P14621 [2-99]
STAQSLKSVDYEVFGRVQGVCFRMYTEDEARKIGVVGWVKNTSKGTVTGQVQGPEDKVNSMKSWLSKVGSPSSRIDRTNFSNEKTI
SKLEYSNFSIRY

>Human Kerato-epithelin Q15582 [24-683]
GPAKSPYQLVLQHSRLRGRQHGPNVCAVQKVIGTNRKYFTNCKQWYQRKICGKSTVISYECCPGYEKVPGEKGCPAALPLSNLYET
LGVVGSTTTQLYTDRTEKLRPEMEG**PGSFTIFAPSNEAWASLPAEVLDSLV**SNVNIELLNALRYHMVGRRVLTDELKHGMTLTSMY
QNSNIQIHHYPNGIVTVNCARLLKADH**HATNGVVHLIDKVISTITNNIQQIIEIEDTFETLRAAVAASGLNTML**EGNGQYTLLAPT

NEAFEKIPSETLNRILGDPEALRDLLNNHILKSAMCAEAIVAGLSVETLEGTTLEVGCSGDMLTINGKAIISNKD**ILATNGVIHYI**
DELLIPDSAKTLFELAAESDVSTAIDLFRQAGLGNHLSGSERLTLLAPLNSVFKDGTPPIDAHTRNLLRNHIIKDQLASKYLYHGQ
TLETLGGKKLRVFVYRNSLCIENSCIAAHDK**RGRYGTLFTMDRVLTPPMGTVMDVLKGDNRFSMLVAAIQSAGLTET**LNREGVYTV
FAPTNEAFRALPPRERSRLLGDAKELANILKYHIGDEILVSGGIGALVRLKSLQGDKLEVSLKNNVVSVNKEPVAEPDIMATNGVV
HVITNVLQPPANRPQERGDELADSALEIFKQASAFSRASQRSVRLAPVYQKLLERMKH

>Human PAPf39 peptide P15309 [248-286]
GIHKQKEKSRLQGGVLVNEILNHMKRATQIPSYKKLIMY

>Human IgGLambda LC amyloidogenic P04209 [1-112]
QSALTQPASVSGSPGQSITISCTGTTSDVGGYDFVSWYQQHPGKAPKLLIYDVNSRPSGISNRFSGSKS**GNTASLTISGLQAEDEA**
DYYCSSFTTTNSRAVFGGGTKLSVLG

>Human A2NI60_HUMANBRE (Fragment)
DIQMTQSPSSLSASVGDRVTITCQA**SQDISDYLIWYQQKLGKA**PNLLIYDASTLETGVPSRFSGSGSGTEYTFTISSLQPEDIATY
YCQQYDDLPYTFGQGTKVEIKR

>Human PMEL P40967 [25-661]
KVPRNQDWLGVSRQLRTKAWNRQLYPEWTEAQRLDCWRGGQVSLKVSNDGPTLIGANASFSIALNFPGSQKVLPDGQVIWVNNTII
NGSQVWGGQPVYPQETDDACIFPDGGPCPSGSWSQKRSFVYVWKTWGQYWQVLGGPVSGLSIGTGRAMLGTHTMEVTVYHRRGSRS
YVPLAHSSSAFTITDQVPFSVSVSQLRALDGGNKHFLRNQPLTFALQLHDPSGYLAEADLSYTWDFGDSSGT**LISRALVVTHTYLE**
PGPVTAQVVLQAAIPLTSCGSSPVPGTTDGHRPTAEAPNTTAGQVPTTEVVGTTPGQAPTAEPSGTTSVQVPTTEVISTAPVQMPT
AESTGMTPEKVPVSEVMGTTLAEMSTPEATGMTPAEVSIVVLSGTTAAQVTTTEWVETTARELPIPEPEGPDASSIMSTESIT**GSL**
GPLLDGTATLRLVKRQVPLDCVLYRYGSFSVTLDIVQGIESAEILQAVPSGEGDAFELTVSCQGGLPKEACMEISSPGCQPPAQRL
CQPVLPSPACQLVLHQILKGGSGTYCLNVSLA**DTNSLAVVSTQLIMPGQEA**GL**GQVPLIVGILLVLMAVVLASLIYRRRLMKQDF**S
VPQLPHSSSHWLRLPRIFCSCPIGENSPLLSGQQV

>Human Natriureticpeptides A P01160 [124-151]
SLRRSSCFGGRMDRIGAQSGLGCNSFRY

>Human Fibrinogen alpha P02671 [36-866]
GPRVVERHQSACKDSDWPFCSDEDWNYKCPS**GCRMKGLIDEVNQDFTNRINKLKNSLFEYQKNNK**DSHSLTTNIMEILRGDFSSAN
NRDNTYNRVSED**LRSRIEVLKRKVIEKVQHIQLLQKNVRAQLVDMKRLEVDIDIKIRSCRGSCS**RALAREVDLKDYEDQQKQLEQV
IAKDLLPSRDRQHLPLIKMKPVPDLVPGNFKSQLQKVPPEWKALTDMPQMRMELERPGGNEITRGGSTSYGTGSETESPRNPSSAG
SWNSGSSGPGSTGNRNPGSSGTGGTATWKPGSSGPGSTGSWNSGSSGTGSTGNQNPGSPRPGSTGTWNPGSSERGSAGHWTSESSV
SGSTGQWHSESGSFRPDSPGSGNARPNNPDWGTFEEVSGNVSPGTRREYHTEKLVTSKGDKELRTGKEKVTSGSTTTTRRSCSKTV
TKTVIGPDGHKEVTKEVVTSEDGSDCPEAMDLGTLSGIGTLDGFRHRHPDEAAFFDTASTGKTFPGFFSPMLGEFVSETESRGSES
GIFTNTKESSSHHPGIAEFPSRGKSSSYSKQFTSSTSYNRGDSTFESKSYKMADEAGSEADHEGTHSTKRGHAKSRPVRDCDDVLQ
THPSGTQSGIFNIKLPGSSKIFSVYCDQETSLGGWLLIQQRMDGSLNFNRTWQDYKRGFGSLNDEG**EGEFWLGNDYLHLLTQRGSV**
LRVELEDWAGNEAYAEYHFRVGSEAEGYALQVSSYEGTAGDALIEGSVEEGAEYTSHNNMQFSTFDRDADQWEENCAEVYGGGWWY
NNCQAANLNGIYYPGGSYDPRNNSPYEIENGVVWVSFRGADYSLRAVRMKIRPLVTQ

>Human Huntingtin P42858 [1-3142]
MATLEKLMKAFESLKSFQQQQQQQQQQQQQQQQQQQQQPPPPPPPPPPQLPQPPPQAQPLLPQPQPPPPPPPPPPPGPAVAEEPLH
RPKKELSATKKDRVNHCLTICENIVAQSVRNSPEFQKLLGIAMELFLLCSDDAESDVRMVADECLNKVIKALMDSNLPRLQLELYK
EIKKNGAPRSLRAALWRFAELAHLVRPQKCRPYLVNLLPCLTRTSKRPEESVQETLAAAVPKIMASFGNFANDNEIKVLLKAFIAN
LKSSSPTIRRTAAGSAVSICQ**HSRRTQYFYSWLLNVLLGLLVPVEDEHSTLLILGVLLTLRYLVPLLQQQVKDTSLKG**SFGVTRKE
MEVSPSAEQLVQVYELTLHHTQHQD**HNVVTGALELLQQLFRTP**PPELLQTLTAVGGIGQLTAAKEESGGRSRSGSIVELIAGGGSS
CSPVLSRKQKGKVLLGEEEALEDDSESRSDVSSSALTASVKDEISGELAASSGVSTPGSAGHDIITEQPRSQHTLQADSVDLASCD
LTSSATDGDEEDILSHSSSQVSAVPSDPAMDLNDGTQASSPISDSSQTTTEGPDSAVTPSDSSEIVLDGTDNQYLGLQIGQ**PQDED**
EEATGILPDEASEAFRNSSMALQQAHLLKNMSHCRQPSDSSVDKFVLRDEATEPGDQENKPCRIKGDIGQST**DDDSAPLVHCVRLL**
SASFLLTGGKNVLVPDRDVRVSVKALALSCVGAAVALHPESFFSKLYKVPLDTT**EYPEEQYVSDILNYIDHGDPQVRGATAILCGT**
LICSILSRSRFHVGDWMGTIRTLTGNTFSLADCIPLLRKTLKDESSVTCKLACT**AVRNCVMSLCSSSYSELGLQLIIDVLTLRNSS**
YWLVRTELLETLAEIDFRLVSFLEAKAENLHRGAHHYTGLLKL**QERVLNNVVIHLLGDEDPRVRHVAAASLIRLVPKLFYKCDQGQ**
ADPVVAVARDQSSVYLKLLMHE**TQPPSHFSVSTITRIYRGYNLLPSITDVTMENNLSRVIAAVSHEL**ITSTTRALTFGCCEALCLL
STAFPVCIWSLGWHCGVPPLSASDESRKSCTVGMATMILTLLSSAWFPLDLSA**HQDALILAGNLLAASAPKSLRSSWAS**EEEANPA
ATKQEEVWPALGDRALVPMVEQLFSHLLKVINICAHVLDDVAPGPAIKAALPSLTNPPSLSPIRRKGKEKEPGEQASVPLSPKKGS
EASAASRQSDTSGPVTTSK**SSSLGSFYHLPSYLKLHDVLKATHANYKVTLDLQ**NSTEKFGGFLRSALDVLSQILELATLQDIGKCV
EE**ILGYLKSCFSREPMMATVCVQQLLKTLFGT**NLASQFDGLSSNPSKSQGRAQRLGSSSVRPGLYHCFMAPYTHFTQALADASLR
NMVQAEQENDTSGWFDVLQKVSTQLKTNLTSVTKNRADK**NAIHNHIRLFEPLVIKALKQYTTTTCVQLQKQVLDLLAQLVQLRVNY**
CLLDS**DQVFIGFVLKQFEYIEVGQFRESEAIIPNIFFFLVLLSYERYHSKQIIGIPKIIQLCDGIM**ASGRKAVTHA**IPALQPIVHD**
LFVLRGTNKADAGKE**LETQKEVVVSMLLRLIQYHQVLEMFILVLQQCHKENEDKWKRLSRQIADIILPMLAKQQMHIDSHEALGVL**
NTLFEILAPSSLRPVDMLLRSMFVTPNTMASVSTVQLWISGILAILRVLISQSTEDIVLSRIQELSFSPYLISCTVINRLRDGDST

STLEEHSEGKQI**KNLPEETFSRFLLQLVGILLEDIVTKQLKVE**MSEQQHTFY**CQELGTLLMCLIHIFKSGMFRRITAAATRLFRSD**
G**CGGSFYTLDSLNLRARSMITTHPALVLLWCQILLLVNHTDYRWWAE**VQQTPKRHSLSSTKLLSPQMSGEEEDSDLAAKLGMCNRE
IVRRGALILFCDYVCQNLHDSEHLTWLIVNHIQDLISLSHEPPVQDFISAVHRNSAASGLFIQAIQSRCENLSTPTMLKKTLQCLE
GIHLSQSGAVLTLYV**DRLLCTPFRVLARMVDILACRRVEMLLAANLQSS**MAQLPMEELNRIQEYLQSSGLAQRHQRLYSLLDRFRL
STMQDSLSPSPPVSSHPLDGDGHVSLETVSPDKDWYVHLVKSQCWTRSDSALLEGAELVNRIPAEDMNAFMMNSEFNLSLLAPCLS
LGMSEISGGQKSALFEAAREVTLARVSGTVQQLPAVHHVFQPELPAEPAAYWSKLND**LFGDAALYQSLPTLARALAQYLVVVSKLP**
SHLHLPPEK**EKDIVKFVVATLEALSWHLIHEQIPLSLDLQ**AGLDCCCLALQLPGLWSVVSSTEFVTHACSLIYCVHFILEAVAVQP
GEQLLSPERRTNTPKAISEEEEEVDPNTQNPKYITAACEMVAEMVESLQSVLALGHKR**NSGVPAFLTPLLRNIIISLARLPLVNSY**
TRVPPLVWKLGWSPKPGGDFGTAFPEIPVE**FLQEKEVFKEFIYRINTLGWTSRT**QFEETWATLLGVLVTQPLVMEQEESPPEEDTE
RTQINVLAVQAITSLVLSAMTVPVAGNPAVSCLEQQPRNKPLKALDTRF**GRKLSIIRGIVEQEIQAMVSK**RENIATHHLYQAWDPV
PSLSPATTGALISHEKLLLQINPERELGSMSYKLGQVSIHSVWLGNSITPLREEEWDEEEEEEADAPAPSSPPTSPVNSRKHRA**GV**
DIHSCSQFLLELYSRWILPSSSARRTPAILISEVVRSLLVVSDLFTERNQFELMYVTLTELRRVHPSEDEILAQYLVPATCKAAAV
LGMDKAVAEPVSRLLESTLRSSHLPSRVGALHGVLYVLECDLLDDTAKQLIPVISDYLLSNLKGIAH**CVNIHSQQHVLVMCATAFY**
LIENYPLDVGPEFSASIIQMCGVMLSGSEESTPS**IIYHCALRGLERLLLSEQLSRLDAESLVKLSVDRVNVHSPHR**AMAALGLMLT
CMYTGK**EKVSPGRTSDPNPAAPDSESVIVAMERVSVLFDRIRKGFPCEARVVARILPQFLDDFFPPQDIMN**KVIGEFLSNQQPYPQ
FMATVVYKVFQTLHSTGQSSMVRDWVMLSLSNFTQRAPVAMATWSLCFFVSASTSPWVAAILPHVISRMGKLEQVDVNLFCLVAT
DFYRHQIEEELDRRAFQSVLEVVAAPGSPYHRLLTCLRNVHKVTTC

>Human Ig kappa chain V-I regionMev (Bence-Jones protein) P01612 [1-109]
DVQMTQSPSSLSASVGDRVIITCRASQSSVDYLNWYQQKPGKAPKLLIFDTSNLQSGVPSRFSGGRSGTDFTLTISSLQPDDFATY
YCQQSYTNPEVTFGGGTTVDIKR

>Somatostatin-14
AGCKNFFWKTFTSC

>AL-09 Amyloidogenic Kappa1 Bence Jones protein
ATDIQMTQSPSSLSASVGDRVTITCQASQDINNYLIWYQQKPGQAPKLLIYDASTLETGVPSRFSGSGSGTEFTFTISSLQPEDLA
TYHCQQYDNLPYTFGQGTKLEIK

>Amyloidogenic Ig Lambda LC V-IV region BAU3rC34Y variant
SYELTQPPSVSVSPGQTASITCSGDKLGDKYAYWYQQKPGQSPVLVIYQDSKRPSGIPERFSGSNSGNTATLTISGTQAMDEADYY
CQAWDSSTAVVFGGGTKLTVL

>Amyloidogenic Kappa-4IgVL, REC
DIVMTQSPDSLAVSPGERATINCKSSQNLLDSSFDTNTLAWYQQKPGQPPKLLIYWASSRESGVPDRFSG**SGSGTDFTLTISSLQA**
EDVAVYYCQQYYSTPPTFGGGTKVEIKR

>IgLC dimer Sea
ETALTQPASVSGSPGQSITVSCTGVSSIVGSYNLVSWYQQHPGKAPKLLTYEVNKRPSGVSDRFSGSKSGNSASLTISGLQAEDEA
DYYCSSYDGSSTSVVFGGGTKLTVLGQPKAAPSVTLFPPSSEELQANKATLVCLISDFYPGAVTVAWKADSSPVKAGVETTKPS**KQ**
SNNKYAASSYLSLTPEQWKSHRSYSCQVTHEGSTVEKTVAPTAC

>AL-103P95A mutant
MRAKLLGIVLTTPIAISSFASTDIQMTQSPSSLSASVGDRVTITCQASQDISNYLIWYQQKPGKAPKLLIYDASNLETGVPSRFSG
SGSGTDFTFTISSLQPEDIATYYCQQYHNLPYTFGPGTKLEIK

>Ig LC lambda 3 3mJL2
SYELMQPPSVSVSPGQTARITCSGDALPKQYAYWYQQKPGQAPVLVIYKDSERPSGIPERFSGSSSGTTVTLTISGVQAEDEADYY
CQSADSSGTYVVFGGGTKLTVL

>Ig LC lambda 3 3rCW variant
SYELTQPPSVSVSPGQTASITCSGDKLGDKYAYWYQQKPGQSPVLVIYQDSKRPSGIPERFSGSNSGNTATLTISGTQAM
DEADYYCQAADSSTAVVFGGGTKLTVL

>6aJL2-R24G amyloidogenic Light chain
NFMLTQPHSVSESPGKTVTISCTGSSGSIASNYVQWYQQRPGSSPTTVIYEDNQRPSGVPDRFSGSIDSSSNSASLTISGLKTEDE
ADYYCQSYDSSNHVVFGGGTKLTVL

>6aJL2 amyloidogenic Light chain
NFMLTQPHSVSESPGKTVTISCTRSSGSIASNYVQWYQQRPGSSPTTVIYEDNQRPSGVPDRFSGSIDSSSNSASLTISGLKTEDE
ADYYCQSYDSSNHVVFGGGTKLTVL

>AL-09H87Y Ig LC

STDIQMTQSPSSLSASVGDRVTITCQASQDINNYLIWYQQKPGQAPKLLIYDASTLETGVPSRFSGSGSGTEFTFTISSLQPEDLA
TYYCQQYDNLPYTFGQGTKLEIKR

Figure S2: Amino acid sequences of the 44 amyloidogenic proteins annotated with predicted APRs and TcAIERs. The APRs were predicted using TANGO (Yellow background) and WALTZ (Turquoise background). In the cases, where the two predictions overlap, the yellow background was retained. If the overlap is partial, the backgrounds for only the additional residues were color in Turquoise. The predictions for TcAIERs were subdivided into fidel (Underlined) and promiscuous (Bold Red Font).

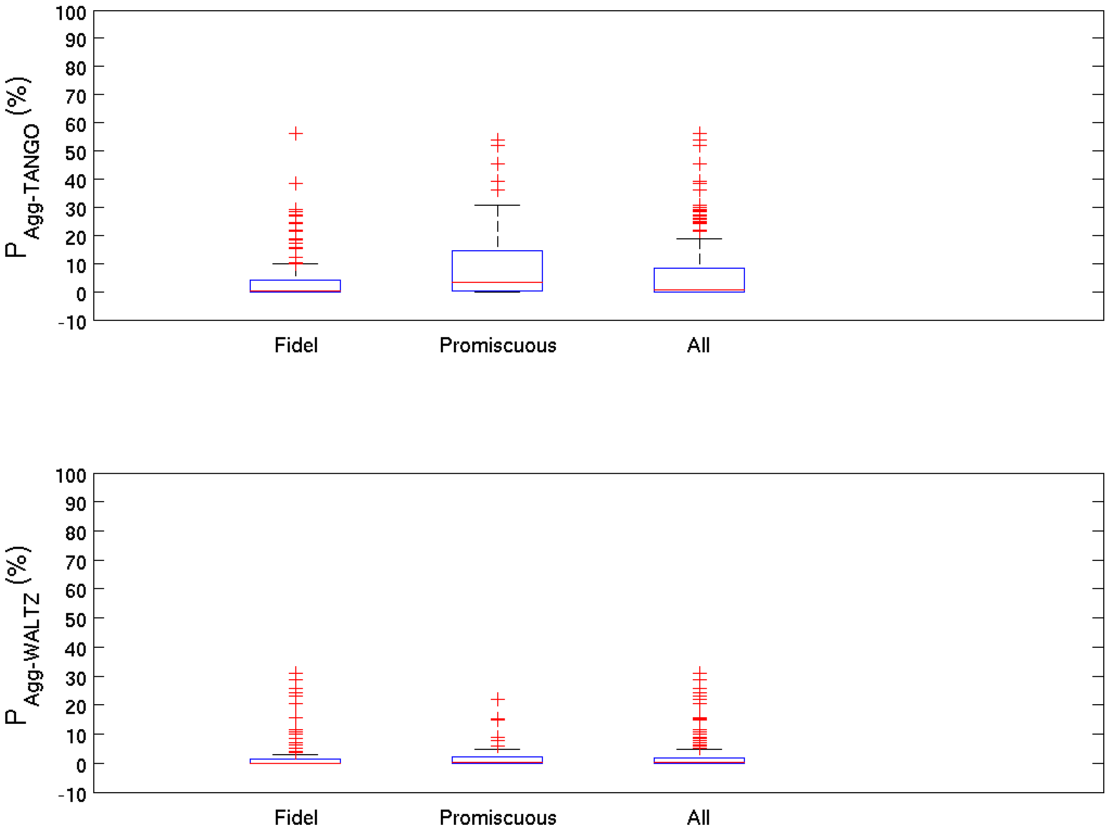

Figure S3: Aggregation propensities of strongly predicted T-cell autoimmune epitopes in the amyloidogenic proteins calculated using TANGO and WALTZ. The 285 predicted autoimmune epitopes overlap in the protein sequences and were consolidated into 194 autoimmune epitope regions. These 194 autoimmune epitope regions were further classified as fidel (130) and promiscuous (64). This figure shows that several predicted T-cell autoimmune epitopes are significantly aggregation prone (Aggregation propensity ≥10%).

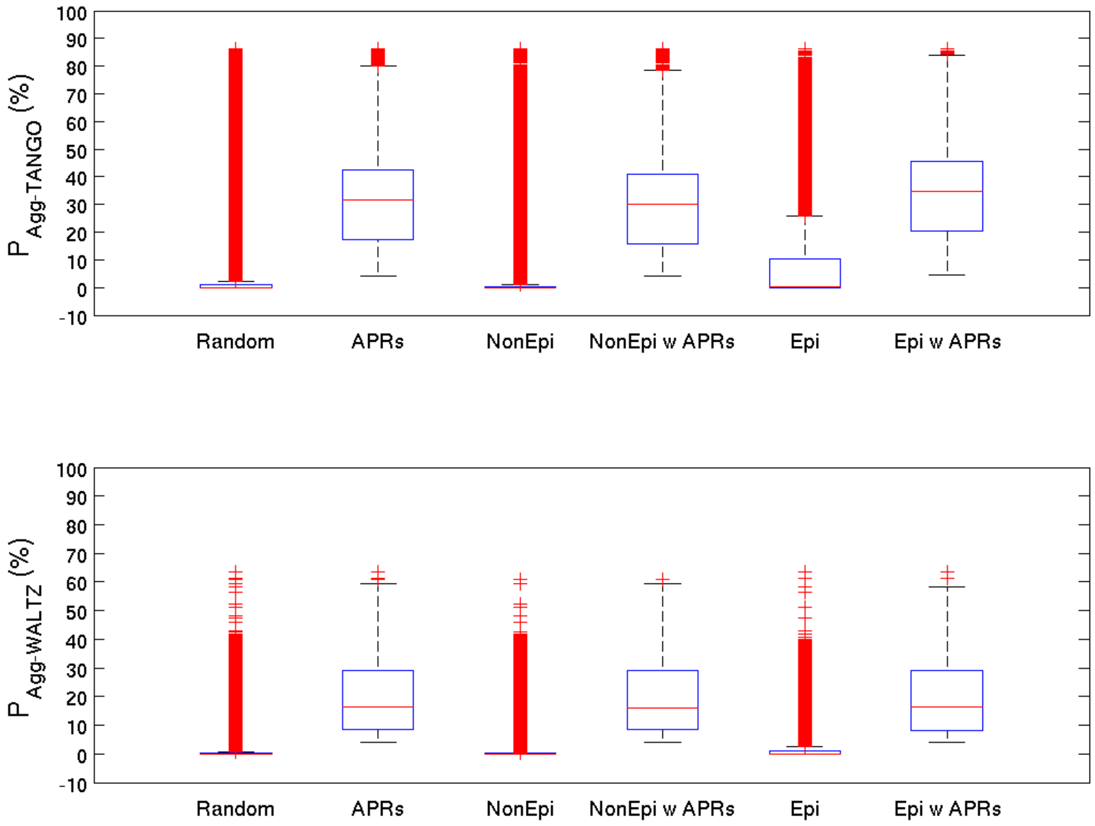

Figure S4: Box and Whisker plots showing aggregation propensities in different datasets of randomly generated 15-residues long peptides. 'Random' stands for all 100,000 randomly generated peptides. 'APRs' stands for peptides that contain TANGO (top panel, 12,179 peptides) and WALTZ (bottom panel, 9,582 peptides) predicted aggregation prone regions. 'NonEpi' stands for 83,616 peptides that were not predicted to be T-cell autoimmune epitopes in this study. 'NonEpis w APRs' stands for non-epitope peptides that contain TANGO (top panel, 8,559 peptides) or WALTZ (bottom panel, 7,261 peptides) predicted aggregation prone regions. 'Epi' stands for 16,384 peptides that are predicted to be T-cell autoimmune epitopes. Finally, 'Epi w APRs' stands for epitopes that contain TANGO (top panel, 3,620 peptides) or WALTZ (bottom panel, 2,321 peptides) predicted aggregation prone regions.
